# In situ prebiotics: enzymatic release of galacto-rhamnogalacturonan from potato pulp in vivo in the gastrointestinal tract of the weaning piglet

**DOI:** 10.1186/s13568-015-0152-1

**Published:** 2015-10-16

**Authors:** Mikael Lenz Strube, Tim Kåre Jensen, Anne Strunge Meyer, Mette Boye

**Affiliations:** National Veterinary Institute, Technical University of Denmark, Frederiksberg, Denmark; Department of Chemical Engineering, Technical University of Denmark, Lyngby, Denmark

**Keywords:** Pectin, Potato, Enzymes, Pectin lyase, Polygalacturonase

## Abstract

Prebiotics may be efficient for prevention of intestinal infections in humans and animals by increasing the levels of beneficial bacteria and thereby improving gut health. Using purified prebiotics may however not be cost-effective in the livestock production industry. Instead, prebiotic fibres may be released directly in the gastro-intestinal tract by feeding enzymes with a suitable substrate and allowing the prebiotics to be produced in situ. Using low doses, 0.03 % enzyme-to-substrate ratio, of the enzymes pectin lyase and polygalacturonase in combination with potato pulp, a low-value industrial by-product, we show that high molecular weight galacto-rhamnogalacturonan can be solubilized in the stomach of weaning piglets. The release of this fiber is in the order of 22–38 % of the theoretical amount, achieved within 20 min. The catalysis takes place mainly in the stomach of the animal and is then followed by distribution through the small intestines. To our knowledge, this is the first paper describing targeted production of prebiotics in an animal model.

## Introduction

Prebiotics, e.g. indigestible fibers fermented by the intestinal microbiota, such as polymers of fructose or galactose, have shown promising results in increasing the intestinal population of beneficial bacteria such as bacteria belonging to the genera *Lactobacillus* and *Bifidobacterium* (Roberfroid et al. 2010). Since strains of *Bifidobacterium* and *Lactobacillus* can combat infectious bacteria (Servin 2004; Liévin-Le Moal and Servin 2014), it is plausible that increasing the levels of these microorganisms through feeding of prebiotics would improve the resilience towards infections in susceptible animals, such as the weaning pig.

It has recently been shown that high molecular weight galacto-rhamnogalacturonan can increase the levels of *Lactobacillus* in in vitro fermentations of piglet ileum content (Strube et al. 2015) and increase the levels of *Bifidobacteria* in in vitro fermentations of human feces (Thomassen et al. 2011b). Galacto-rhamnogalacturonan can be extracted enzymatically from low-value potato pulp (Thomassen et al. 2011a; Ravn and Meyer 2014), a waste product from the starch industry available in large quantities (Meyer et al. 2009). Although extraction of these carbohydrates in an industrial setting may be feasible for human use, the cost-efficiency can potentially be increased by carrying out the enzymatic catalysis in the gastro-intestinal tract (GIT) of the animal by feeding a supplement of the substrate and enzymes. Although a main function of GIT is to digest proteins, and hence would be expected to inactivate enzymes through hydrochloric acid and proteases, the digestive system of the young pig is less harsh than in the adult (Strube et al. 2013), making these animals prime candidates for in situ production of beneficial prebiotic fibers.

The carbohydrate released from potato pulp by enzyme action consists of the pectinaceous domain galacto-rhamnogalacturonan (GRG), which is an alternating backbone of rhamnose and galacturonic acid substituted with galactose, and, to a smaller extent, arabinose chains (Mohnen 2008; Harholt et al. 2010). The pectin in potato pulp is tightly bound to the cellulose/lignin matrix and is not water soluble unless treated with pectinases (Strube et al. 2015).

In this study, the feasibility as well as the time frame of in situ enzyme catalysis of potato pulp in vivo in weaning piglets is investigated.

## Materials and methods

All reagents were of analytical grade and purchased from Sigma-Aldrich (Sigma-Aldrich, Steinheim, Germany) unless otherwise noted.

### Characterization of pectinolytic enzymes

Pectin lyase (PL) and polygalacturonase (PG) were produced as monocomponent enzymes by fermentations as described in de Silva et al. (Silva et al. 2011) using *Pichia pastoris* clones transformed with the PL gene AN2569.2 and the PG gene AN4372.2, both from *Aspergillus nidulans,* as described in Bauer et al. (Bauer et al. 2006). The protein concentrations in the enzyme solutions following sterile filtration and ultrafiltration were determined by the bicinchoninic acid assay with bovine serum albumin as a standard as described by the manufacturer (Thermo Fisher Scientific, Rockford, IL).

PL activity was assayed by incubating 100 µL of enzyme sample, diluted 10 times in 200 mM *N*-Cyclohexyl-2-aminoethanesulfonic acid (CHES), pH 8.6, with 100 µL 1 g/L citrus pectin (Sigma-Aldrich, Steinhein, Germany) in 200 mM CHES, pH 8.6, at 37 °C in triplicate. The reaction was followed for 60 min in an Infinite200 Microplate Reader (Tecan, Salzburg, Austria) by recording the absorbance at 235 nm. A standard curve of 0–1.5 mg/L PL was used to express the amount of enzyme in the samples as percentages of the fed amount. A measured activity corresponding to 0.012 mg enzyme, corresponding to 2 % (w/w) of the fed amount, was considered a positive presence of enzyme in the given section.

PG was assayed by a modified ruthenium red assay (Torres et al. 2011; Ortiz et al. 2014). Briefly, samples were diluted twice in 200 mM acetate buffer (pH 4.5) and 60 µL diluted sample was added to 20 µL 4 g/L polygalacturonic acid (Sigma-Aldrich) in acetate buffer at pH 4.5 at 600 RPM and 20 °C in triplicate. After 60 min of incubation, the enzymatic reaction was stopped by adding 920 µL 100 mg/mL ruthenium red followed by mixing at 1000 RPM for 5 min and centrifugation for 5 min at 5000*g*. Controls were made by addition of diluted sample after addition of ruthenium red. A standard curve of 0–1.375 mg/L PG was used to express the amount of enzyme in the samples as percentages of the fed amount. A measured activity corresponding to 0.012 mg enzyme, corresponding to 2 % of the fed amount, was considered a positive presence of enzyme in the given section.

Pepsin in the stomach supernatant was assayed by incubating 50 µL supernatant in 250 µL 0.06 M HCl with 20 g/L hemoglobin in triplicate. The reaction was run for 37 °C at 900 RPM and terminated after 60 min with 500 µL of 5 % w/v trichloracetic acid. After 10 min of mixing, the samples were centrifuged for 2 min at 14,000*g* and 100 µL of the supernatant was measured at 280 nm in an Infinite200 microplate reader (Tecan, Salzburg, Austria). Controls were made by adding the trichloracetic acid before the gastric sample. Units were defined as nmol tyrosine released per minute using an extinction coefficient of 1250 M^−1^cm^−1^. Pepsin concentration was calculated by comparing the activity to a standard curve of commercial pepsin [P7125, #SLBB6557V] (Sigma-Aldrich).

### Animals

Twenty pigs were purchased from a commercial farm in Denmark at 30 days of age, 2 days after weaning. The animals were of mixed breed, had been given a wheat based creep feed since 7 days of age, had not been given antibiotics or growth promoters and weighed ~7 kg. The pigs were fed a commercial weaning feed, Porkido™, 24 h before the start of the experiments and were not fed on the day of the experiment.

All handling of animals was performed by trained personnel and veterinarians, fulfilling the regulations from the Danish Ministry of Justice.

### Experimental design

FiberBind (KMC a.m.b.a, Brande, Denmark), the dried residue following starch removal from potatoes, was ground to ~300 µm particle size and 2000 mg was weighed off into a syringe. To avoid catalysis outside the animals, all reagents were kept on ice: 5 min before feeding, 19 mL of ice-cold water was drawn into the syringe and mixed thoroughly on ice. Immediately before use, 1 mL of 0.6 g/L of each PL and PG was drawn into the syringe, resulting in a final concentration of 0.03 g/L of each enzyme e.g. an enzyme-to-substrate ratio of 0.03 % (w/w). Control animals received no enzyme. The reaction was initiated by orally placing a rubber tube in stomach of each animal by which they were given the mixture. Animals were sacrificed by an overdose of pentobarbital and jugular bleeding in duplicate at 20, 40, 60, 120 and 180 min after feeding. Immediately after death, the pigs were necropsied and the entire contents of the stomach and small intestine was extracted and put on dry ice, followed by storage at −20º C until further analysis. Before extraction of the content from the small intestine, the entire intestine was laid out and divided into three equally long sections; the contents of which is hence referred to as Duo, Jeje and Ile.

### Determination of reaction progress

All handling of samples was done on ice. Sample weight was noted, followed by measurement of pH using a pH-meter and dry matter by overnight heating at 105 °C. Subsequently, samples were spun at 21,000*g* for 10 min at 4 °C. Filtration was avoided due to occasional low sample volume. The supernatant was then measured for the activity of PL and PG as well as pepsin activity. The polymeric carbohydrate content in the remaining supernatant was precipitated by addition of isopropanol to a final concentration of 70 % (v/v) after which it was centrifuged at 5000*g* for 5 min. The supernatant was discarded and the pellet was dried in a vacuum drier and weighed.

The chemical characteristics of GRG are the monosaccharides rhamnose, galacturonic acid and galactose as well as a molecular size larger than 100 kDa. As such, the measure of reaction progress was then the amount of the monosaccharides rhamnose, galactose and galacturonic acid as well as the amount of carbohydrate with a molecular mass larger than 100 kDa (CH100) in the IPA-precipitated residue. Rhamnose and galacturonic acid in particular are unique to pectin.

### Carbohydrate composition

Monosaccharide composition analysis was done by a modified NREL sulphuric acid hydrolysis (Sluiter et al. 2011). In brief, ~5 mg of finely ground carbohydrate sample was added to 300 µL 72 % sulfuric acid, followed by 60 min incubation in a water bath at 30 °C. Next, 8.4 mL of water was added and samples were autoclaved at 121 °C for 60 min. Monosaccharides were then quantified on a HPAEC-PAD system fitted with a Dionex CarboPac PA1 analytical column (2 × 250 mm) combined with a CarboPac PA1 precolumn (4 × 50 mm) by eluting with 0.25–500 mM NaOH at 1 mL/min (Ravn et al. 2015). Fucose, rhamnose, arabinose, galactose, glucose, xylose, galacturonic acid were included as standards. Glucosamine and galactosamine were included to account for monosaccharides from the host. Monosacharides concentrations were calculated from the standard curves, giving the concentration in the hydrolyzed sample and, in turn, the total mass of monosaccharide in the intestinal content.

Size determinations were done by dissolving carbohydrate samples at 3 g/L in 0.1 M sodium acetate, pH 6, with 0.02 % (w/v) sodium azide followed by filtering with a 0.22 µm filter. Samples where then injected in Shodex OHpak SB-806 HQ (8.0 × 300 mm) column (Showa Denko KK, Kawasaki, Japan), and eluted with 0.1 M sodium acetate (pH 6). The injection volume was 100 µL and the flow rate was 0.5 mL/min at 30 °C on a system consisting of a P680 HPLC pump and an ASI-100 automated sample injector using a refractive index detector Shodex RI-101 (Showa Denko KK). Pullulan standards of 1.3, 10, 110, 400 and 800 kDa were used. As the precipitate was not always fully soluble, the concentration of CH100 was calculated from a standard curve of fully soluble GRG. This was then expressed as the mass of CH100 in the intestinal content.

### In vitro reaction kinetics

The action of each enzyme in the stomach was evaluated by an in vitro model of the piglet stomach. 1000 mg of FiberBind (300 µm particle size) was added to 30 mL of water with 0.32 mg/mL pepsin which was adjusted to pH 2 with 475 µL 1 M HCl. This was preheated in a 45 °C water bath for 5 min, after which 0.3 mg of either PG or PL was added. Controls were without any enzyme. To stop reactions, tubes were shaken vigorously on ice and immediately centrifuged for 5 min at 5000*g* at 5 °C. 10 mL of supernatant was then filtered in a Whatman 40 filter and precipitated with 23.3 mL isopropyl alcohol, after which the supernatant was discarded and the pellet was dried in a vacuum drier and weighed. CH100 could be used as the sole reaction parameter, since the in vitro reactions did not contain obscuring compounds such as the in vivo ones.

### Data presentation and statistics

For the in vivo data, means are presented in graphs. Linear regression and squared Pearson correlation coefficients were used to relate rhamnose, galacturonic acid, galactose and CH100 with one another and to examine relationship with incubation time and PG. To ease interpretation, data is generally presented as the cumulative amount in the entire GIT, e.g. the sum of the four sections in each animal. ANCOVA was used to examine the effect of time and enzyme addition on the accumulated release of the four main parameters. When appropriate, the data is also expressed as a percentage of the theoretically available amount, calculated from Strube et al. (Strube et al. 2015).

In vitro data was fitted with a modified Monod equation of the form.

$${\text{CH1}}00 = {\text{a}} + ({\text{b }}*{\text{ t}}/({\text{c}} + {\text{t}})),$$

where t is time, a is the value of CH100 at t = 0, b is the maximum value with value at t = 0 subtracted, c is the time at which half of the maximum release is attained.

## Results

### Animal characteristics

All mixtures were successfully given at all time-points. Generally, substantial variation within the animals was observed, likely owing to biological variation and inherent difficulties in classifying the exact position of the content in the small intestine. Moreover, several sections were empty in some animals. In spite of the 24 h fasting, 16 out of 20 animals had a dry matter content in the GIT of more than the 2000 mg administered in the feeding tubes. This accumulation was mainly found in the stomach, suggesting delayed clearance of fed in a subset of animals.

The pH did not differ between enzyme-treated or control animals in any of the sections, and was 2.37 ± 0.51 in the stomach, whereas in the small intestine, it ranged between 5.50 and 8.30. One animal (enzyme group, 180 min) had a stenosis in the ileum near the ileocecal valve, resulting in abnormal amounts of fluids in the small intestine and a pH of 3 in the duodenum.

### Solubilization of galacto-rhamnogalacturonan

**Fig. 1:**
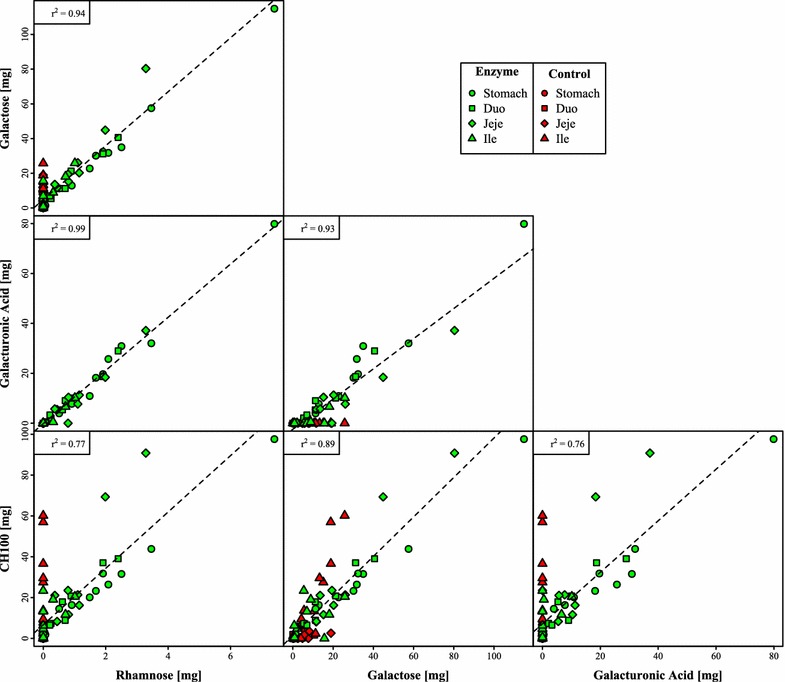
Matrix plot of variables chemically associated with galacto-rhamnogalacturonan, e.g. galactose, galacturonic acid, rhamnose and CH100. All collected samples are included, e.g. from all sections at all time-points for both enzyme- and control-treated animals. Each point is the total mass of the given variable in the given sample. r^2^ is the squared Pearson correlation of the two variables in each plot. *CH100* carbohydrate with a molecular mass larger than 100 kDa

The mass of released CH100, galactose, galacturonic acid and rhamnose was highly correlated in the animals fed enzyme (Fig. 1); suggesting that released carbohydrate was GRG from potato pulp, as this domain is large and composed of a backbone of rhamnose and galacturonic acid highly substituted by galactose. GRG is from here on used as an umbrella term for these four parameters. This correlations was not found in the control animals, mainly because these samples were almost entirely devoid of both rhamnose and galacturonic acid. A substantial amount of galactose and CH100 was, however, found in the ileal samples of some control animals, but this is presumably of mucosal origin since this was accompanied by high levels of the mucosal carbohydrates galactosamine, glucosamine and fucose.

There was little association between the incubation period and the amount of GRG solubilized (data not shown), although there was a tendency of the GRG to move terminally with incubation time. Notably, the stomach of enzyme-treated animals was devoid of rhamnose at all time-points apart from 20 min, and in contrast, the levels in the jejenum increased with time.

**Fig. 2:**
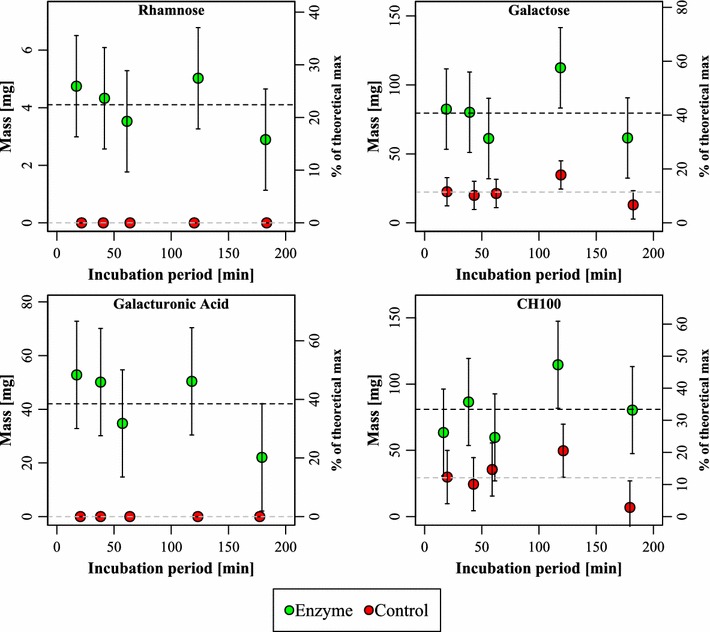
The cumulative amount of solubilized rhamnose, galacturonic acid, galactose and CH100 when summed across all sections of the gastrointestinal tract at each time point, for both enzyme- and control-treated animals. *Vertical lines* are intercepts in the associated linear model. Values are mean ± SEM. On the *right axis*, the mass relative to the theoretically maximal amount is depicted. *CH100* carbohydrate with a molecular mass larger than 100 kDa

When summed over all four sections in each animal, incubation time was not associated with a higher release of rhamnose, galacturonic acid, galactose or CH100 (*p* value for slope > 0.05) as seen in Fig. 2. There was however a highly significant effect of enzyme addition in general (p < 0.05). The total solubilization of GRG was between 22 and 38 % judging by the release of rhamnose and galacturonic acid, respectively, relative to the theoretical maximum amount. The lack of a time dependency may suggest that the reaction has progressed to completion within the initial 20 min and the GRG is only being distributed through the GIT beyond this. A certain microbial digestion of GRG from e.g. galactanases or RG-1 lyases produced by native bacteria in the ileum is also possible.

### Enzyme activity

PG activity was observed, on average at 10 % of the initial dose, in all stomach samples but one (120 min) of the enzyme-treated animals, but not in the small intestines. This may suggest that this enzyme is less affected by low pH, but is susceptible to proteases present in the small intestine. Seeing as GRG is released in the stomach, this may be less relevant. Interestingly, PG activity was found in low amounts in 4 control animals. PG activity correlated well with the amount of rhamnose, galacturonic acid, galactose and CH100 in the enzyme-treated animals, but only in the stomach samples (r^2^ = 0.65–0.75).

In contrast, PL was not measurable in the stomach but was sporadically active in the small intestine, suggesting that this enzyme may survive both the stomach and small intestinal environment. It does not seem to be relevant for the release of GRG, as this seem to happen in the stomach environment. PL was not found in control animals.

### In vitro stomach model

**Fig. 3:**
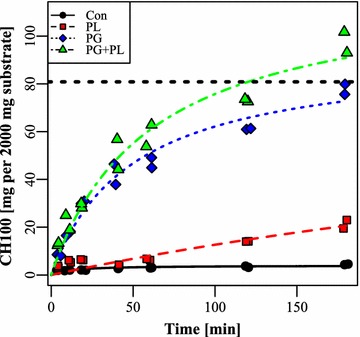
Released CH100 with PG, PL or both in combination in an in vitro reactor emulating stomach conditions, e.g. pH 2 and pepsin at 0.32 mg/mL. Values are scaled to the same mass of initial substrate as in the in vivo data to allow for direct comparison. *Lines* were fitted with a Monod equation. The *vertical line* corresponds to the average of CH100 in the entire gastrointestinal tract of the enzyme-treated animals. *CH100* carbohydrate with a molecular mass larger than 100 kDa, *PG* polygalacturonase, *PL* pectin lyase

To test the hypothesis that the majority of GRG-release is achieved rapidly in the stomach conditions, the release of GRG in a simulated stomach with low pH and pepsin was investigated (Fig. 3). Very little CH100 was released when no enzyme was added. With PL added, a small increase in release was observed, up to 20 mg after 180 min. When PG was added, however, 10 mg was released within 5 min, and almost 80 mg was released after 180 min. The combination of PG and PL was comparable to only PG, but resulted in a release of 100 mg at 180 min. The release appears to be facilitated by PG-activity and the mass of the released carbohydrate is comparable to what was observed in vivo.

## Discussion

In this work, the feasibility of enzymatically releasing GRG from potato pulp in the GIT of the weanling pig was evaluated. The animals showed high biological variability, especially evident by the high variation in stomach content of dry matter despite the 24 h fasting before the start of the experiment. This residual material is however in agreement with reports from the literature such as Gregory et al. (1990), where a large meal was not expected to be completely cleared after 24 h in growing pigs (Gregory et al. 1990). In newly weaned pigs Snoeck et al. (2004) showed stomach retention of >20 % of a meal at 24 h post-feeding and incomplete clearance even after 50 h (Snoeck et al. 2004). Furthermore, chemical analysis was somewhat obscured by the presence of residual foodstuffs and mucousal matter, which unfortunately, is high in galactose in mammals (Allen et al. 1998). In contrast, it is low in rhamnose and galacturonic acid (Allen et al. 1998; Giraud and Naismith 2000), making these well suited for following the release of GRG.

Targeted in situ production of prebiotics has not been thoroughly investigated, although the generation of carbohydrate oligomers from non-starch polysaccharides by feed carbohydrases is probable if not inevitable. Several authors have already hypothesized upon this phenomena by measuring secondary outcomes such as gut bacterial composition and generation of bacterial metabolites (SCFAs) in pigs fed with supplementary enzymes (Vahjen et al. 2007; Kiarie et al. 2007; Pauly et al. 2011; Murphy et al. 2012), but these have shown limited effect and focusing mainly on xylanase and glucanases. Studies investigating in situ oligosaccharide generation mainly revolve around xylan and include Courtin et al. (2008) and Bindelle et al. (2011), although none of these systematically focus on the generation in vivo. Bindelle et al. managed to change the composition of the microbiota of in vitro fermentations of wheat based diets including feed enzymes (Bindelle et al. 2011), whereas Courtin et al. observed comparably positive effects on feed conversion rate from purified arabinoxylan and a wheat based diet supplemented with xylanase (Courtin et al. 2008). A similar approach was explored by Damen et al. (Damen et al. 2012a, 2012b), in which xylanase was used to release arabinoxylan in bread dough during the kneading process.

The present work builds upon earlier data, where it was shown that GRG could be solubilized from dried potato pulp by action of PL and PG in a simulated porcine in vitro GIT reactor (Strube et al. 2015), as it had been shown for an industrial process (Thomassen et al. 2011b; Ravn et al. 2015). Here, a miniscule amount of enzyme, 0.03 % of the substrate mass, was shown to release GRG in 1 h in stomach conditions plus 1 h in small intestinal conditions. In the present work, we tested this in vivo by feeding a mixture of enzyme and dried potato pulp to newly weaned piglets and following the release of GRG throughout 180 min. Monosaccharide analysis and size exclusion showed that substantial amounts of rhamnose, galacturonic acid, galactose and high molecular weight carbohydrate (>100 kDa) was indeed released, and that these measures were highly correlated (Fig. 1), suggesting that GRG was in fact released as this is the chemical signature of potato GRG (Mohnen 2008; Ravn et al. 2015). Surprisingly, it appeared that most, if not all, GRG was released within the first 20 min in the stomach, and was then subsequently distributed through the GIT. Analysis of residual enzyme revealed that PG was active in the stomach samples, but not in other sections of the GIT tract, whereas PL was much less stable. Residual PG activity furthermore correlated well with amount of GRG in the stomach samples, suggesting that PG is the prime driver of GRG solubilization. We verified this by emulating the stomach conditions in an in vitro reactor using the measured pH values and pepsin activities and observed a fast release of CH100 mainly by the action of PG. PL was not efficient in this system, owing to the fact that this enzyme has a pH-optimum in the alkali range, whereas PG is active in acidic conditions (Thomassen et al. 2011a). Pectin lyase may possibly be omitted in future work. The overall dose of enzyme could be increased, as could the doses used, 0.03 % w/w is indeed very low.

In conclusion, the catalytic release of GRG from potato pulp by endogenous enzymes is possible in the weaning piglet, and this catalysis is primarily driven by PG activity in the stomach. Given that GRG has shown prebiotic potential (Thomassen et al. 2011b; Strube et al. 2015), it is proposed that feeding piglets potato pulp and pectinolytic enzymes would result in an improved microbiota and improved gut health, potentially increasing resilience towards intestinal infections.
